# Value of growth arrest lines for predicting treatment effect on children with distal tibial epiphysis fractures

**DOI:** 10.3389/fped.2023.1040801

**Published:** 2023-02-08

**Authors:** Sisheng Wang, Shaoluan Zheng, Qi Liu, Chengyun Wang, Maosheng Liu, Lianbin Su

**Affiliations:** ^1^ Department of Pediatric Orthopedics, First Affiliated Hospital of Xiamen University, Xiamen, China; ^2^ Department of Plastic and Reconstructive Surgery, Zhongshan Hospital (Xiamen), Fudan University, Xiamen, China

**Keywords:** growth arrest lines, epiphyseal fracture, bone bridge, premature epiphyseal closure, prognosis

## Abstract

**Objective:**

This study aims to explore whether growth arrest lines can predict epiphyseal fracture healing.

**Method:**

The data of 234 children with distal tibial epiphysis fractures treated in our hospital from February 2014 to February 2022 were retrospectively analyzed. Imaging data were examined to record epiphyseal grade, fracture type, and the time to appearance of growth arrest lines. Follow-up data were retrieved to record treatment results (i.e., malunion, premature closure, or bone bridge formation).

**Results:**

There was a significant difference in the time to appearance of growth arrest lines between patients with epiphyseal grade 0–1 and grade 2–3 (*P* < 0.05) and between patients with normal healing and patients with a bone bridge (*P* < 0.05). Among patients with normal healing, there were no significant differences in the time to appearance of growth arrest lines between men and women and between patients with and without surgery (*P* > 0.05). There was a significant difference in the time to appearance of growth arrest lines between patients with different Salter–Harris fracture types (*P* < 0.05).

**Conclusion:**

For patients with epiphyseal grade 0–1, the time to appearance of growth arrest lines could be useful for predicting the treatment result of a distal tibial epiphyseal fracture.

## Introduction

Growth arrest lines, also known as growth resumption lines, Harris lines, or Park lines, are high-density rings of sclerosis seen on radiographs at the metaphysis of long bones of children with disturbance of epiphyseal growth. These lines form due to alternating cycles of osseous growth arrest and growth resumption. Growth arrest lines, which can be detected on radiographs and CT images, are usually the result of malnutrition, disease, or trauma in children but may also occur due to alternating periods of normal growth and growth surge (1). Growth arrest lines are common during the healing process of a tibial epiphyseal fracture. Caterini et al. (2) suggested that the appearance and morphology of the line could be used to predict abnormal epiphyseal growth after ankle fracture in children. However, there has been no research so far on its predictive value. The aim of this retrospective study was to determine whether growth arrest lines could be used to predict treatment results after tibial fractures in children.

## Materials and methods

### Materials

The institutional review board of our hospital approved this study with a waiver of the need for informed consent.

The hospital records were reviewed to identify all children treated for distal tibial epiphyseal fractures at the Department of Orthopedics of the First Affiliated Hospital of Xiamen University from February 2014 to February 2022. Patients were included in this study if they (1) were ≤14 years old at the time of treatment, (2) had been followed up for more than 4 months, and (3) had been followed up until treatment finished when complications of the fracture were found. The exclusion criteria were (1) pathological fracture, (2) hormone treatment for other diseases, (3) open injury or craniocerebral injury, or (4) endocrine disease. A total of 234 children (132 boys and 102 girls; age range, 8–14 years) met these criteria; 129 children underwent operative treatment.

### Radiography method

Radiographs were obtained using a machine with a tube current of 200–500 mA and a tube voltage of 80–100 kV. Anteroposterior and lateral films of the ankle joint were obtained at a tube voltage of 50–55 kV. Radiographs were obtained with the injured limb extended at the knee and the contralateral limb flexed. The long axis of the tibia was kept parallel to the long axis of the film. The foot is in dorsiflexion and rotates inward by 10°–15°. The center of the film was 1 cm above the midpoint of the ankle joint, and the knee-to-film distance was 75–90 cm.

### Image analysis

**Figure 1:**
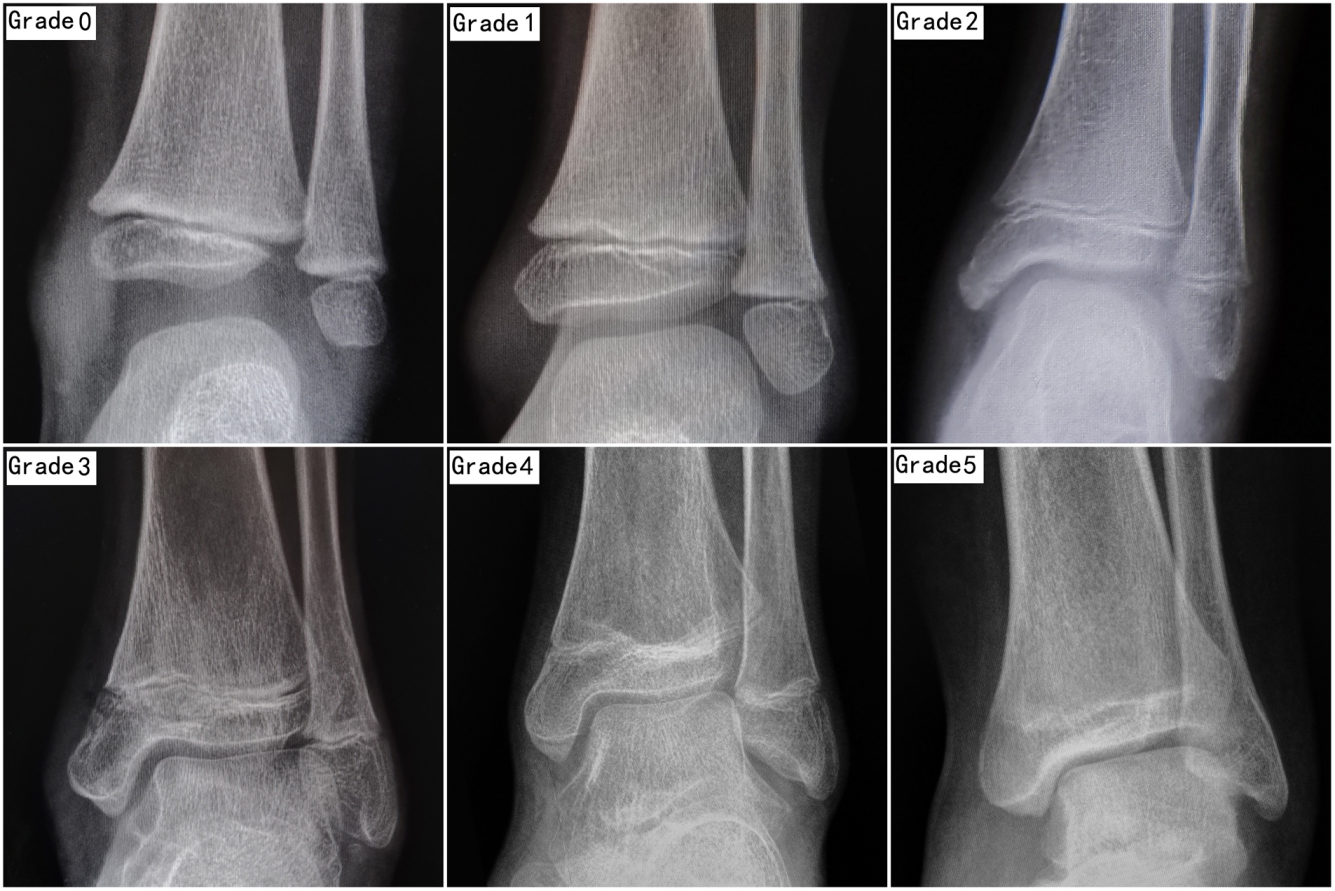
X-ray of grades of tibial epiphysis development.

Two experienced pediatric orthopedic surgeons independently analyzed the radiographs and graded the fractures according to the Salter–Harris method; if CT had been performed, the grading was based on CT images. The degree of development of the distal tibial epiphysis was evaluated according to the criteria proposed by Zhu et al. (3), who classified tibial epiphysis development into six types (Table 1, Figure 1). Table 2 shows the baseline characteristics of the patients.

**Table 1:** Grades of tibial epiphysis development.

Grade	Content
Grade 0	Epiphyseal cartilage space is wide and clear. The maximum transverse diameter of the secondary ossification center is smaller than that of the metaphysis, and the medial malleolus ossification has not been found
Grade 1	Epiphyseal cartilage space is wide and clear. The maximum transverse diameter of the secondary ossification center is slightly wider than that of the metaphysis, and the medial malleolus ossification could be found
Grade 2	The closure of the metaphysis has begun. The epiphyseal cartilage space becomes narrow and fuzzy. It can be seen that the trabecula passes through epiphyseal cartilage space
Grade 3	The epiphysis is partially closed, and the closure range is less than 1/2. The closure mostly begins in the middle
Grade 4	Most of the metaphyses were closed, and the closed range was more than 1/2. There is still space on one or both sides of the metaphysis
Grade 5	All metaphyses were closed, and the epiphysis line remained or disappeared

**Table 2:** Baseline characteristics of patients.

	Type 0	Type 1	Type 2	Type 3
Sex, *n*				
Male	8	99	19	6
Female	10	65	20	7
Age, years, mean ± SD				
Male	7.75 ± 1.80	11.19 ± 1.46	13.16 ± 0.49	13.50 ± 0.55
Female	7.80 ± 1.03	10.65 ± 1.35	12.65 ± 1.14	13.85 ± 0.38
Treatment, *n*				
Surgical	6	90	23	10
Nonsurgical	12	74	16	3
Salter–Harris type, *n*				
I	7	5	0	0
II	11	138	29	10
III	0	11	4	1
IV	0	10	6	2

There were no patients of type 4 and type 5 in the study. So, there is no frame of type 4 and type 5.

Whether and when growth arrest lines appeared were recorded. Growth arrest lines were recorded as being present only if there was an agreement between both pediatric orthopedic surgeons. The time of appearance of the growth arrest line was recorded as the time from the injury to the follow-up date on which the growth arrest line was first recognized on imaging. Malunion, premature epiphyseal closure, or bone bridge formation during follow-up were noted.

### Statistical methods

Measurement data were summarized as means ± standard deviation and analyzed using the *t*-test. Count data were expressed as rates and analyzed using the chi-square test. SPSS 25.0 (IBM Corp., Armonk, NY, United States) was used for data analysis. Statistical significance was set at *P* < 0.05.

## Results

Table 3 shows the occurrence rate of the growth arrest line in children with different types of epiphyseal development. The rate was not significantly different between type 0 and type 1 (*P* > 0.05). However, there was a significant difference in rate between type 0–1 and type 2–3 (*P* < 0.05). The occurrence rate was not significantly different between the two sexes (*P* > 0.05).

**Table 3:** Occurrence rate of growth arrest lines in patients with different epiphyseal types.

	Type 0	Type 1	Type 2	Type 3
Male	100%	95.96%	0%	0%
Female	100%	95.38%	0%	0%

Children with epiphyseal type 0–1 and growth arrest lines were divided into two groups according to the treatment result: a normal healing group and a bone bridge formation group. Meanwhile, children without growth arrest lines were divided into a normal healing group and an epiphyseal premature closure group (Table 4).

**Table 4:** Treatment result of patients with epiphyseal type 0–1.

	With growth arrest line		Without growth arrest line		Total
	Normal healing	Bone bridge formation	Normal healing	Epiphyseal premature closure	
Males, *n*	101	2	1	3	107
Female, *n*	71	1	1	2	75
Total	172	3	2	5	182

The occurrence time of the growth arrest line in the normal healing group was 43.74 ± 8.28 days, and the time in the bone bridge formation group was 88.00 ± 4.00 days. There was no statistical difference between the two groups (*P* < 0.05).

Among patients with normal healing, the time to appearance of the growth arrest line was comparable between patients of different sexes and between patients receiving different treatments (*P* > 0.05). There was a significant difference in the mean time to appearance of the growth arrest line among patients with different Salter–Harris types (*P* < 0.05). Table 5 shows the details.

**Table 5:** Time to appearance of growth arrest line in normal healing patients.

	Type 0	Type 1
Sex		
Male	37.38 ± 6.82	48.86 ± 8.95
Female	38.50 ± 6.29	49.00 ± 8.70
Treatment		
Surgical	40.66 ± 6.56	49.99 ± 9.92
Nonsurgical	36.66 ± 5.0	47.68 ± 7.25
Salter–Harris grade		
I	38.00 ± 5.54	39.20 ± 6.42
II	38.00 ± 7.10	48.47 ± 6.84
III	—	48.44 ± 6.91
IV	—	48.20 ± 6.26

Data in columns are the mean number of days ± standard deviation.

## Discussion

The growth arrest line is a sign of epiphyseal growth disturbance. Various factors that cause temporary bone growth disturbance and stunted growth may induce the appearance of growth arrest lines (4, 5). One such factor is a fracture. In the healing process of long bone shaft fractures in children, hematoma formation and fibrocartilaginous callus formation will initially cause temporary bone growth arrest. Then, in the bony callus formation stage, regenerated blood vessels and local osteogenic stimulation will cause rapid growth of the epiphysis. The growth rate on the affected side will exceed that on the opposite side for a short period. Epiphyseal calcium salt deposition in the growth arrest period results in the appearance of the growth arrest line on radiographs—a high-density line in the metaphysis, close to and parallel to the epiphysis. This high-density line will gradually move away from the epiphysis during the growth period and can therefore be used to monitor the growth and development of the epiphysis after an injury. In this study, we reviewed cases of distal tibial epiphyseal fractures treated at our hospital to evaluate the value of the growth arrest line for predicting the probability of epiphyseal deformity after injury.

The shape of long bone epiphyses varies between individuals, but, in all cases, the epiphyseal plate of the distal tibia appears as a line in both anteroposterior and lateral radiographs. Observing whether the growth arrest line is parallel to the epiphyseal plate can provide useful information.

A distal tibial fracture is relatively common in children. According to Audigé et al. (6), lower leg fracture accounts for 75% of all long bone fractures in the lower extremity and fracture of distal metaphysis and epiphysis accounts for 43% of them. Therefore, for this study, we focused on distal tibial epiphyseal fractures.

Growth and development vary greatly among children. Bone age estimation becomes less precise as age advances, with a mean absolute error ranging between 11 and 21 months (7), so age is an inaccurate parameter for assessing growth and development potential. In the clinic, it is impractical to obtain additional radiographs for assessing bone age. For patients with distal tibial epiphyseal fracture, the epiphyseal development grade of the distal tibia can be directly observed on anteroposterior radiographs of the ankle. Therefore, in this study, we used the epiphyseal development grade as an index of the growth potential of the tibial epiphysis.

We found that, in patients with epiphyseal development grades 0 and 1, growth arrest lines appeared in most patients with normal healing. However, no growth arrest line appeared in patients with epiphyseal development grade 2 and above. This is to be expected. Grade 2 epiphyseal stage marks the beginning of epiphyseal closure. From then on, the epiphysis begins to calcify gradually, and there is no major growth of the extremities; thus, the growth arrest line will not form. In this period, the treatment of the fracture is the same as in adults.

**Figure 2:**
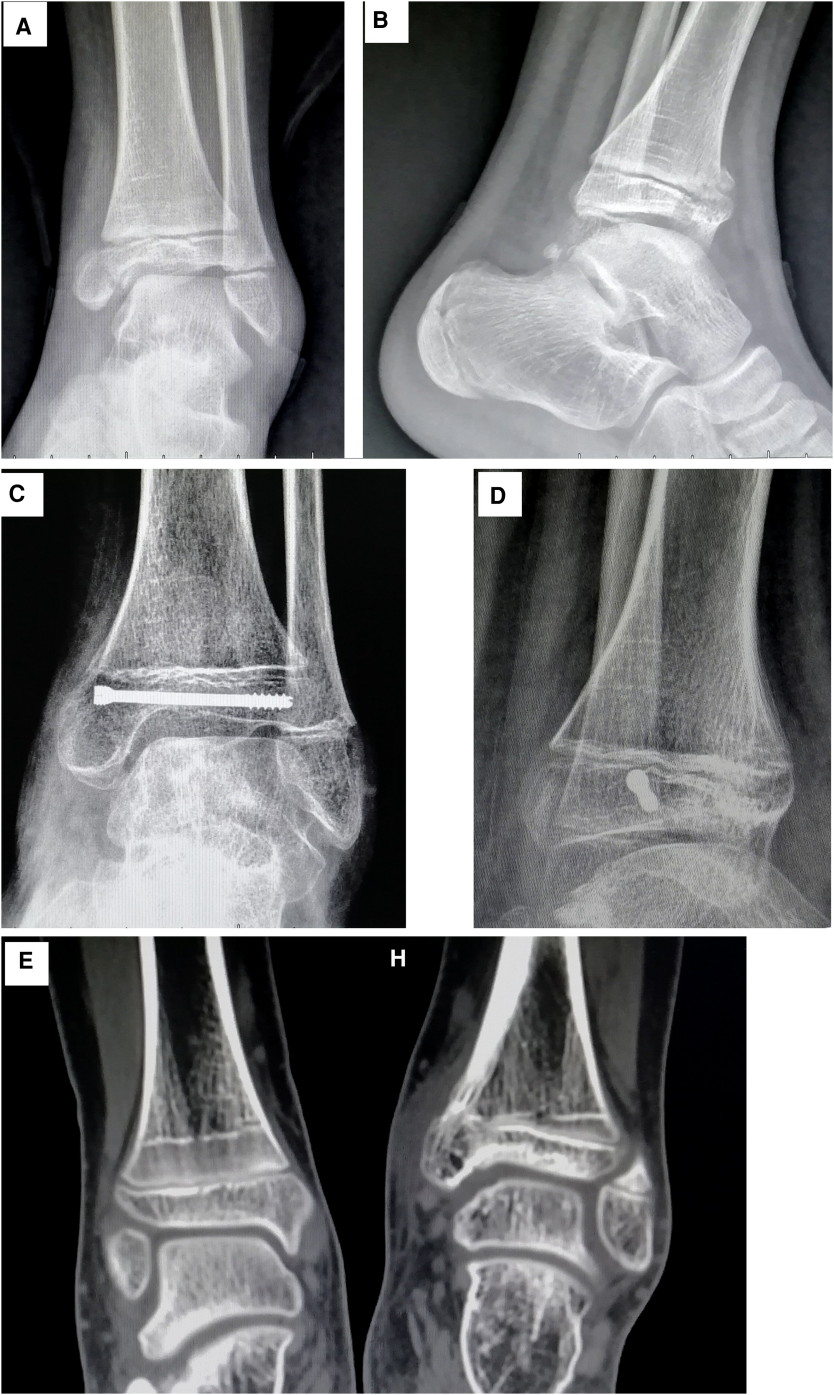
(**A**) Radiographs and CT images of a 12-year-old boy. The anteroposterior view shows a tibial epiphyseal fracture on the left side. (**B**) Lateral view showing that the fracture line crosses the epiphyseal plate (Salter–Harris type IV fracture). (**C**) Radiograph taken after 90 days of follow-up showing the growth arrest line. Formation of the medial bone bridge also can be seen. (**D**) Lateral view showing the bone bridge in the front of the epiphysis. (**E**) CT scan of bilateral ankle joints showing that the growth arrest line of the healthy side is parallel to the epiphysis, whereas the growth arrest line of the affected side forms an angle with the epiphysis. The apex of the angle is at the medial bone bridge.

**Figure 3:**
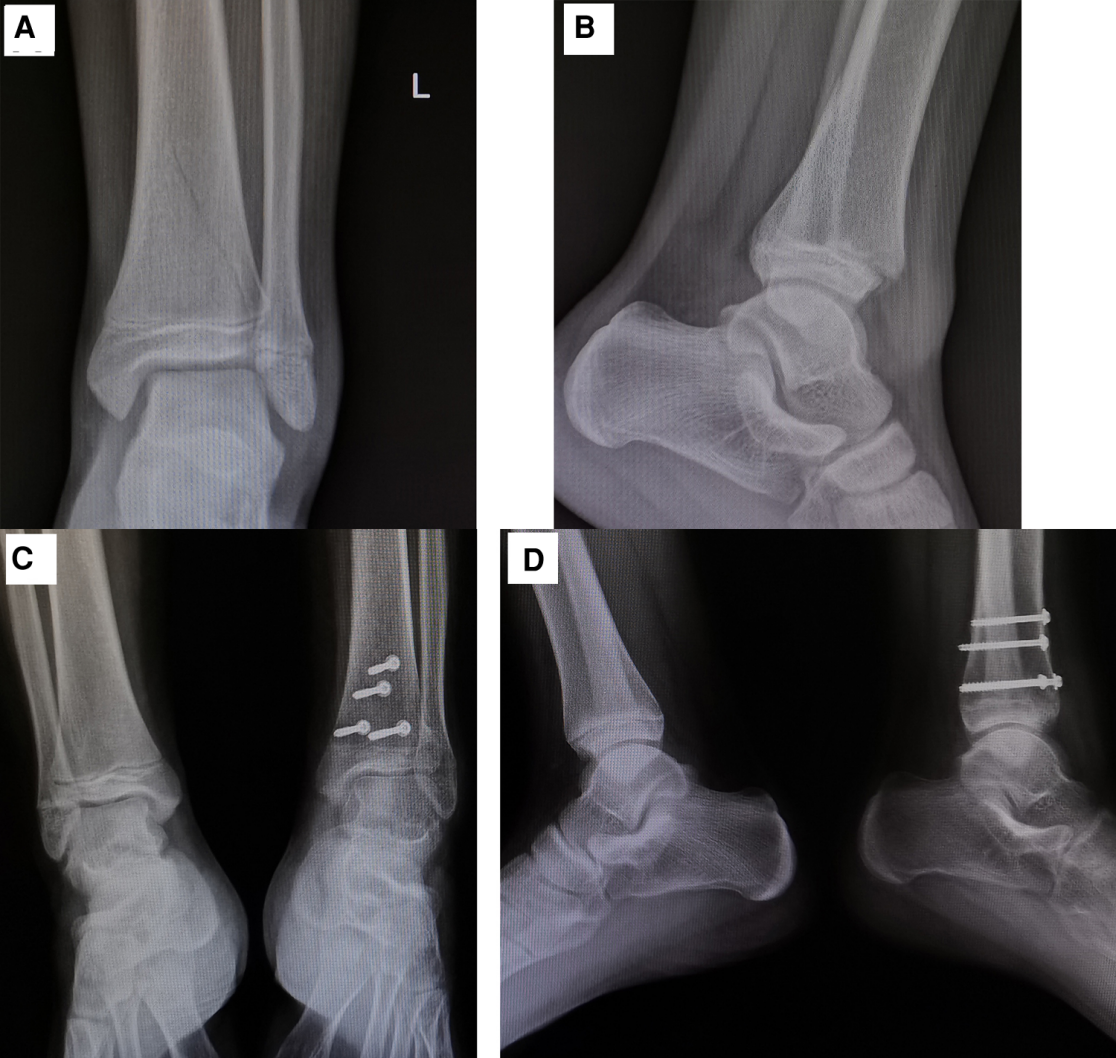
(**A**) Radiographs of a 13-year-old boy. The anteroposterior view of the left ankle joint shows the fracture line extending to the epiphysis. (**B**) Lateral view showing the fracture line extending from the posterior side of the tibial shaft to the anterior side of the epiphysis. The distal part of the epiphysis is displaced backward. This was classified as a Salter–Harris type II fracture. (**C**) At 6 months after the injury, the anteroposterior view of bilateral ankle joints shows no growth arrest lines. The epiphyseal plate of the affected side was blurred, while the epiphyseal plate of the contralateral side was still visible. This was judged as premature epiphyseal closure. (**D**) Lateral view of bilateral ankle joints showing also premature closure of the epiphyseal plate of the affected side.

In most patients with epiphyseal development grade 0, growth arrest lines appeared within 5–6 weeks of the injury; the mean time to appearance of these lines was 37.38 ± 6.82 days in males and 38.50 ± 6.29 days in females. In patients with epiphyseal development grade 1, the lines generally appeared within 7–8 weeks (mean time: 48.86 ± 8.95 days in males and 49.00 ± 8.70 days in females). In children with normal healing, the growth arrest line runs parallel to the epiphysis, and its distance from the epiphysis gradually increases with bone growth. In patients with epiphyseal grades 0 and 1, those without growth arrest lines can be divided into three types according to the healing pattern: normal healing, bone bridge formation, and premature epiphyseal closure. In our study, only 2/174 (1.15%) children without growth arrest lines had normal healing. In patients with bone bridge formation, the appearance of the growth arrest line was delayed. As the child grows, the growth arrest line and the epiphyseal line gradually form an angle. The apex of the angle is where the bone bridge forms (Figure 2). For patients with premature epiphyseal closure, the epiphyseal plate will gradually become blurred and calcified. In teenage patients, if it is difficult to judge whether there is premature epiphyseal closure or normal closure, a radiograph of the contralateral ankle joint can help (Figure 3).

Among patients with normal healing, those with epiphyseal development grade 0 had an earlier appearance of growth arrest line than patients with epiphyseal development grade 1; this may be related to the stronger growth potential of the epiphysis in the former. However, in each epiphyseal development grade group, the time to appearance of the growth arrest line was not associated with sex or the mode of treatment (surgery vs. no surgery). Tomaszewska and Psonak also found that, although growth arrest lines are more frequently seen in males than in females, the difference was not statistically significant (8). We found an association between the presence of a growth arrest line and fracture type. However, in general, the time to appearance of the growth arrest line was significantly shorter in patients with normal healing (43.74 ± 8.28 days) than in patients with bone bridge formation (88.00 ± 4.00 days). Thus, patients with a significantly delayed appearance of growth arrest line should be suspected of having a bone bridge or premature epiphyseal closure.

In this study, all patients with epiphyseal development grade 0 had Salter–Harris type I and type II fractures. The time to appearance of the growth arrest line was in the range of 5–6 weeks (type I: 38.00 ± 5.54 days, type II: 38.00 ± 7.10 days). Among patients with epiphyseal development grade 1, the time to appearance of growth arrest line in those with Salter–Harris type I fracture was also about 5–6 weeks (39.20 ± 6.42 days); however, in those with Salter–Harris type II, III, and IV fractures, the time to appearance of growth arrest line was about 7–8 weeks (type II: 48.47 ± 6.84 days, type III: 48.44 ± 6.91 days, type IV: 48.20 ± 6.26 days).

This study has some limitations. The epiphyseal development classification does not fully reflect skeletal maturity. The age range of epiphyseal development grade 1 is wide. So the skeletal maturity in this group can vary widely. Salter–Harris type I fractures are completely contained within the epiphysis; there is no associated bone fragment. This type of fracture usually occurs when there is relatively little epiphyseal ossification, which means that the distal epiphysis is practically a single piece of cartilage. In the study by Hofsli et al. (9), the mean age of patients with Salter–Harris type I fracture was 10 years. Therefore, in fact, the skeletal maturity of patients with Salter–Harris type I fracture in the epiphyseal development grade 1 group may be more similar to epiphyseal development grade 0 than other fracture types in the epiphyseal development grade 1 group. This is a disadvantage of using epiphyseal development grade to judge epiphyseal growth potential. Another limitation of this study is that clinical follow-up is often affected by the personal factors of patients, and there is a long interval between follow-up visits, usually ∼2 weeks. So, it is not possible to accurately identify the time to appearance of the growth arrest line through follow-up. However, this study showed that even in patients with different fracture types, the occurrence time of growth arrest line in normal healing patients is far shorter than that in bone bridge formation patients. Moreover, the growth arrest line was observed in most patients with epiphyseal development grades 0 and 1. One previous study in children and adolescents found that the degree of initial displacement was the only significant risk factor for growth arrest after an epiphyseal fracture of the distal tibia (10). However, initial displacement cannot be used to evaluate treatment results. We therefore used the time to appearance of the growth arrest line to evaluate the recovery of epiphyseal fracture.

## Conclusion

For children with epiphyseal fracture of the distal tibia, the time to appearance of growth arrest lines, combined with epiphyseal development grade, can help predict prognosis. If the growth arrest line cannot be seen at 6 weeks in patients with epiphyseal development grade 0 or until 8 weeks in patients with epiphyseal development grade 1, bone bridge formation or premature closure of epiphysis must be suspected, and further inspection should be performed so that prompt treatment can be provided. For patients with epiphyseal development grade 2 and above and without the appearance of the growth arrest line, the treatment is as same as for adults.

## Data Availability

The raw data supporting the conclusions of this article will be made available by the authors, without undue reservation.
